# Chronic dry eye symptoms after LASIK: parallels and lessons to be learned from other persistent post-operative pain disorders

**DOI:** 10.1186/s12990-015-0020-7

**Published:** 2015-04-21

**Authors:** Alexandra E Levitt, Anat Galor, Jayne S Weiss, Elizabeth R Felix, Eden R Martin, Dennis J Patin, Konstantinos D Sarantopoulos, Roy C Levitt

**Affiliations:** Bascom Palmer Eye Institute, University of Miami, 900 NW 17th Street, Miami, FL 33136 USA; Miami Veterans Administration Medical Center, 1201 NW 16th St, Miami, FL 33125 USA; Departments of Ophthalmology, Pathology and Pharmacology, Louisiana State University Health Sciences Center, Louisiana State University Eye Center, New Orleans, LA USA; Department of Physical Medicine and Rehabilitation, University of Miami Miller School of Medicine, Miami, FL USA; John P. Hussman Institute for Human Genomics, University of Miami Miller School of Medicine, Miami, FL USA; John T Macdonald Foundation Department of Human Genetics, University of Miami Miller School of Medicine, Miami, FL USA; Department of Anesthesiology, Perioperative Medicine and Pain Management, University of Miami Miller School of Medicine, Miami, FL USA

**Keywords:** LASIK, Photorefractive keratectomy, Dry eye, Chronic pain, Neuropathic pain, Persistent post-operative pain, Photoallodynia, Peripheral sensitization, Central sensitization

## Abstract

Laser in-situ keratomileusis (LASIK) is a commonly performed surgical procedure used to correct refractive error. LASIK surgery involves cutting a corneal flap and ablating the stroma underneath, with known damage to corneal nerves. Despite this, the epidemiology of persistent pain and other long-term outcomes after LASIK surgery are not well understood. Available data suggest that approximately 20-55% of patients report persistent eye symptoms (generally regarded as at least 6 months post-operation) after LASIK surgery. While it was initially believed that these symptoms were caused by ocular surface dryness, and referred to as “dry eye,” it is now increasingly understood that corneal nerve damage produced by LASIK surgery resembles the pathologic neuroplasticity associated with other forms of persistent post-operative pain. In susceptible patients, these neuropathological changes, including peripheral sensitization, central sensitization, and altered descending modulation, may underlie certain persistent dry eye symptoms after LASIK surgery. This review will focus on the known epidemiology of symptoms after LASIK and discuss mechanisms of persistent post-op pain due to nerve injury that may be relevant to these patients. Potential preventative and treatment options based on approaches used for other forms of persistent post-op pain and their application to LASIK patients are also discussed. Finally, the concept of genetic susceptibility to post-LASIK ocular surface pain is presented.

## Introduction: Ocular nerve injury after LASIK and evidence for persistent ocular pain

Laser in-situ keratomileusis (LASIK) is a common procedure to correct refractive error, with approximately 650,000 cases performed in the US each year [1]. LASIK involves the creation of a superficial flap of corneal epithelium and anterior stroma, which is retracted to allow for the ablation of the underlying tissue, thus correcting refractive error and improving visual acuity. While patients are typically satisfied with outcomes after their procedure [2,3], side effects do occur, mainly in the form of unpleasant ocular sensations described as dryness, burning, and discomfort. [4,5] These symptoms, regarded as components of dry eye, range in severity, but the effects on quality of life may be significant. Utility studies are used to quantify patient experiences and preferences regarding a disease state, and by this metric moderate-to-severe dry eye symptoms are equivalent to moderate-to-severe angina or hospital dialysis, while mild dry eye symptoms might equate with severe migraines [6]. There is a growing literature suggesting that these manifestations of ocular discomfort, commonly described as symptoms of “dry eye,” are better understood as corneal pain. The International Association for the Study of Pain defines pain as “an unpleasant sensory and emotional experience associated with actual or potential tissue damage, or described in terms of such damage,” [7] a broad definition which certainly encompasses the experience of those with the “dry eye” symptoms described above. Furthermore, there is much evidence to support the rationale that the persistent ocular pain that some patients experience following LASIK is a manifestation of corneal neuropathy and the development of central sensitivity.

Qualitatively, the symptoms described following LASIK that have traditionally been conceptualized as “dry eye” are similar to other forms of persistent pain after nerve injury, suggesting that ocular surface pain that develops after LASIK represents a pathological hypersensitivity of the ocular somatosensory nerves. Descriptors frequently endorsed by patients with chronic pain after nerve injury include burning and certain forms of evoked pain (by touch, heat, or cold), pain due to light touch or other innocuous stimuli (allodynia), increased sensitivity to noxious stimuli (hyperalgesia), and expansion of this heightened pain sensitivity beyond the area of initial injury (secondary hyperalgesia) [8,9]. In the eye, these fundamental properties remain with unique elicitors, for example, allodynia to wind, saline eye drops, or light (photoallodynia or photophobia), and the presence of spontaneous pain and dysesthesias (burning). Secondary hyperalgesia also occurs, and patients with corneal neuropathic pain may experience exaggerated conjunctival or scleral pain, in addition to pain along the V1/V2 distribution (including exacerbation of migraine headaches, pain over the face, temporomandibular joint, cheek, etc.) [4,7,10-12]. Additionally, these symptoms of ocular surface pain may be present without detectable disruption of tear film parameters and accompanied by normal or near normal corneal staining patterns, which are used as indicators of corneal surface pathology [11,13].

Both acute and long–term ocular pain and discomfort following LASIK have been reported [6,14,15]. It is suggested that almost all patients have at least mild symptoms after their procedure and that 20-55% of patients have persistent symptoms, defined as symptoms at 6 months or more post-procedure (Table 1) [13,14,16-20].

**Table 1:** **Incidence of chronic dry eye symptoms after refractive surgery**

**Study**	**Procedure**	***N***	**Design**	**Definition**	**Incidence**
Denoyer 2014 [18]	LASIK	60	Prospective series	Use of eye drops at 6 months	43%
De Paiva 2006 [19]	LASIK	35	Prospective randomized (nasal vs. superior hinge)	Fluorescein staining score of 3 or more at 6 months	36.4% (overall)
Shoja 2007 [14]	LASIK	95	Retrospective series	Subjective symptoms at 6 months	20%
Donnenfeld 2003 [20]	LASIK	52	Prospective randomized (nasal vs. superior hinge)	Patients reporting eyes drier than before LASIK at 6 months	31% (overall)
Tuisku 2007 [13]	LASIK	20 cases	Retrospective case-control	Subjective symptoms at 2 - 5 years	55%
Hovanesian 2001 [16]	LASIK and PRK	781	Mailed questionnaire	Subjective symptoms at 6 months or more	44%

LASIK = laser in-situ keratomileusis, PRK = photorefractive keratectomy.

### LASIK induced damage of corneal innervation

#### Corneal neuroanatomy and pain transmission

**Figure 1:**
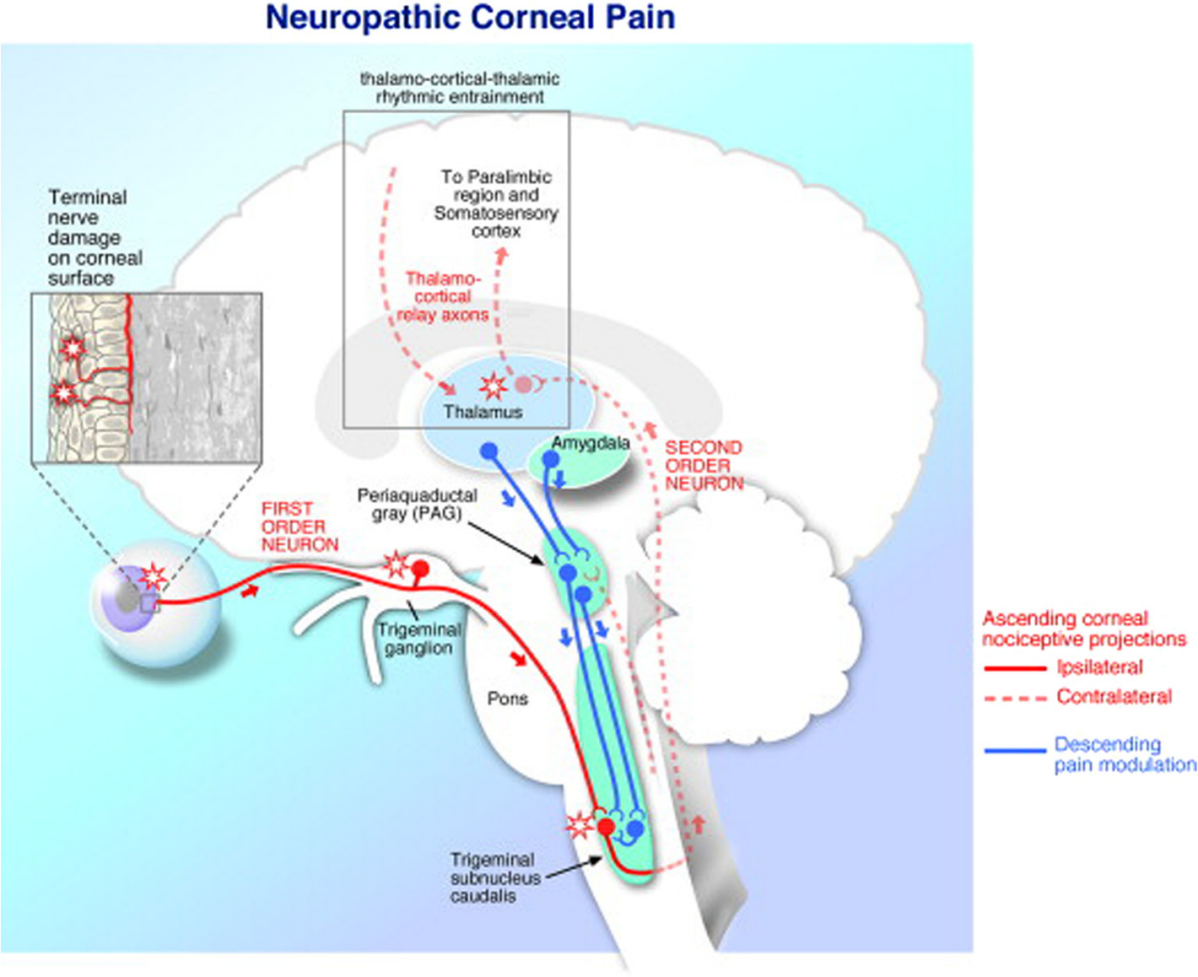
A simplified version of the ocular sensory apparatus. First order neuron (red solid line) with nerve ending in the cornea, cell body in the trigeminal ganglion, and synapse in the subnucleus caudalis. In actuality, however, there are multiple synapses for each nociceptor in the trigeminal subnucleus interpolaris/subnucleus caudalis (Vi/Vc) transition zone, and in the subnucleus caudalis/upper cervical transition zone (Vc/C_1–2_). Second order neurons (red dashed line) decussate and join the contralateral spinothalamic pathways and synapse in the thalamus. Third-order neurons (red dashed line) then relay information to the supra-spinal centers, including the somatosensory cortex. Reproduced with permission from Ocul Surf. 2012 Jan;10(1):2–14.

The cornea is innervated by branches of the nasociliary nerve, a branch of V1, the ophthalmic division of the trigeminal nerve. The nerves enter the periphery in a radial fashion and lose their myelin sheath near the limbus, the junction of the cornea and the sclera [21]. The majority of the corneal nerve fibers (the sub-basal nerve plexus) are located in the anterior third of the stroma and eventually turn 90 degrees to interdigitate between the cells of the superficial epithelium very near to the ocular surface [21]. Corneal nociceptors have their primary cell bodies in the trigeminal ganglion and first synapse in the trigeminal subnucleus interpolaris/subnucleus caudalis (Vi/Vc) transition zone, and in the subnucleus caudalis/upper cervical transition zone (Vc/C_1–2_) [22,23]. Second-order axons originate from the spinal trigeminal nuclear complex, decussate, join the contralateral spinothalamic pathways, and synapse in the thalamus. Third-order neurons then relay information to the supra-spinal centers, including subcortical regions and the somatosensory cortex. Furthermore, descending modulatory pathways exist, originating in various areas of the central nervous system, which modulate the signals of incoming pain and thus pain perception. Corneal nerve damage is expected to alter these ascending and descending pathways representing the substrate of chronic pain (Figure 1).

#### Mechanisms of corneal neurotransmission that may be altered by LASIK

Corneal nociceptors are present in 3 distinct types: roughly 20% are Aδ mechanoreceptors transmitting acute pain; 70% are polymodal, and 10% are C-fiber cold receptors. There is also evidence that there may be another class of receptors that are activated only by local inflammation [24]. The Aδ mechanoreceptor neurons are the fastest conducting, and appear to primarily transmit signals in response to mechanical insults to the ocular surface and may be responsible for the sharp type pain elicited by a foreign body [25]. Polymodal receptor fibers, the most prevalent type, respond to mechanical, thermal, and chemical stimuli as well as endogenous mediators. Pathologic sensitization of this fiber type, likely mediated in part by transient receptor potential (TRP) receptors, including the TRP vanilloid receptor, TRPV1, are believed to play a role in the pathogenesis of neuropathic pain subsequent to corneal nerve injury. Polymodal receptors are also believed to play a role in reflexive tearing subsequent to irritation of the corneal surface [24,25]. Finally, highly sensitive to changes in corneal surface temperature down to 0.5°C, the C-fiber cold thermoreceptors play an important role in maintenance of basal tear secretion (as opposed to the induced tear secretion mediated by polymodal receptors). Tonic firing occurs in the setting of normal corneal/conjunctival temperature of 34-35°C. As the tear film evaporates in the absence of blinking, ocular surface temperature falls by roughly 0.3°C per second and the firing rate increases, eventually stabilizing at a higher frequency proportional to the current corneal temperature [25-27]. As occurs elsewhere in the body, the magnitude of excitatory input is likely dependent on both the frequency of firing and the total volume of activated receptors. Thus, if the overall population of afferent fibers decreases, such as in cases of corneal disease, aging, or perhaps after refractive surgery, cold-dependent basal tearing would be expected to decrease. The thermoregulation of these fibers is largely mediated by TRPM8, a cold-gated ion channel. TRMP8 −/− knock out mice were found to have half the basal tear secretion of their wild-type counterparts, providing evidence for the idea that tonic firing of these receptors plays an important role in maintaining basal tear secretion. These mice had preserved irritation-induced tearing, a phenomenon mediated by polymodal receptors independent of the TRPM8 channel (as discussed above). Besides the three primary classes of corneal nerve fibers, there are also distinct populations defined by neurochemical markers, which are summarized in Table 2, adapted from the text of Shaheen et al. [24].

**Table 2:** **Select neurochemical markers of the cornea, adapted from Shaheen et al., 2014** [24]

**Nerve fiber type**	**Associated markers**
Somatosensory (mechanical)	NF-200
Somatosensory (nociceptive)	
Peptidergic	CGRP
	SP
	PACAP
	Galanin
Non-peptidergic	FRAP
Autonomic	
Sympathetic	NE
	5-HT
	NPY
Parasympathetic	Ach
	VIP
	NPY
	Galanin

NF-200 = neurofilament-200 kDa, CGRP = calcitonin gene-related peptide, SP = substance P, PACAP = pituitary adenylate cyclase activating peptide, FRAP = fluoride-resistant acid phosphatase, NE = norepinephrine, 5-HT = serotonin, NPY = neuropeptide Y, Ach = acetylcholine, VIP = vasoactive intestinal peptide.

#### Persistent sub-basilar plexus damage after LASIK

By virtue of its procedure (cutting a flap and ablating the cornea underneath), LASIK leads to damage to the corneal nerves of the sub-basilar plexus and decreased corneal sensitivity. Immediately post-operatively a majority of patients experience corneal hypoesthesia, which returns to normal values by 6–12 months in some [14,28,29] (but not all [30-36]) studies. However, although corneal sensitivity may normalize, there is potentially permanent damage of the sub-basilar nerve plexus; in studies with up to 5 years post-operative follow up, sub-basilar nerve plexus density has never been demonstrated to return to pre-LASIK levels [13-15,18,37-42]. In a small prospective, non-comparative case series of 48 patients who underwent bilateral LASIK, both corneal and conjunctival sensation were significantly decreased from preoperative levels up to 16 months following the procedure. This hypoesthesia was initially associated with both increased symptom severity scores (dry eye symptoms including pain) and signs of ocular surface pathology (abnormal Schirmer’s, corneal staining). However, although objective signs of ocular surface damage returned to baseline levels by 12 months, symptoms remained significantly elevated from baseline at 16 months (the final time point) [32]. This correlation between corneal hypoesthesia and symptom severity has been reported in multiple studies in both post-LASIK and idiopathic dry eye patients [43-46]. Another study of 20 LASIK patients also reported chronic symptoms without objective corneal abnormalities at 2–5 years post-procedure [13]. The persistence of pain in absence of objective peripheral findings is a pattern consistent with the development of ocular neuropathic pain, and seen in neuropathic pain syndromes elsewhere in the body. Likewise is the seemly paradoxical finding of decreased corneal sensation with findings of increased pain and irritation (e.g., as seen in diabetic and other neuropathies).

### Mechanisms of pathologic neuroplasticity following LASIK induced injury

The development of neuropathic ocular pain is likely a consequence of the neuroplastic changes that occur after ocular nerve injury induced by LASIK. This neuro-remodeling has been well described, and involves peripheral and central pathways as well as descending modulation.

*Peripheral sensitization* is essentially the phenomenon of nociceptor hyperexcitability following injury. Peripheral sensitization involves enhanced ion channel activation and conductance, increased expression of receptors and channels on the cell membrane, and changes in gene expression induced following neurogenic inflammation in response to local injury. Collectively, these changes result in the reduced threshold and increased responsiveness of peripheral nociceptive neurons to the stimulation of their receptive fields [7,47].

Prolonged firing of peripheral nociceptors can also result in neuroplastic changes to the central nervous system, a process known as *central sensitization.* As in the periphery, the bombardment of nociceptive input results in modulation of neurotransmitter release, increases in ion channel and receptor responsiveness and density, and other changes (including alterations in cytosolic signaling pathways), again mediated by inflammatory molecules including cytokines, chemokines, and neuropeptides. Some component of this process has been shown to have an N-methyl-D-aspartate (NMDA) receptor dependent mechanism. Again, the end result is increased responsiveness of nociceptive neurons in the central nervous system to their normal or subthreshold afferent input [7,48]. A hallmark of central sensitization is pain that is disconnected from ongoing peripheral input, a phenomenon commonly seen after LASIK in patients whose symptoms do not mirror objective ocular surface findings, or “pain without stain” [32,49]. The process of central sensitization may initially be reversible, but often becomes permanent [50,51].

The dorsal horn, or in the case of the cornea, the analogous trigeminal nucleus, is an intermediary between the higher central processing centers and peripheral nervous systems, home to the convergence of the circuits critical to the regulation of descending inhibitory modulation, and a key locus in the process of central sensitization [50,51]. The allodynia characteristic of neuropathic pain is thought to originate here with the recruitment of Aβ and C fibers and phenotypic switch from innocuous touch to pain transmission. The dorsal horn/spinal trigeminal nuclei represent an important clinical target, as it is here that opioids, serotonergic and alpha-2 adrenergic agonists, and gabapentinoids exert many of their effects to reduce neuropathic pain by reducing excitatory synaptic neurotransmission (acting presynaptically) and/or by enhancing inhibitory modulation. Additional supraspinal mechanisms modulating pain up to and including the cortex have also been described [47].

### Persistent post-surgical pain after LASIK: What can we learn from other PPP disorders?

Persistent post-surgical pain (PPP) is a known entity that can occur after any surgical intervention, and nerve injury is believed to be the major cause. Typically, this entity is considered to be present in patients with (1) pain that developed after surgery, (2) pain of at least 3–6 months duration (well after sufficient time for healing of those tissues disrupted by surgery), (3) other causes of pain excluded, (4) pain of neuropathic quality (burning, shooting, electric-like), and (5) pain that occurs spontaneously, or in the absence of nociceptor activation [47]. Clinical signs of hyperalgesia (or expansion of receptive fields as in secondary hyperalgesia) or allodynia commonly help to substantiate diagnosis of neuropathic pain. There is substantial overlap in the risk factors and epidemiology of post-LASIK eye pain and somatic PPP syndromes, as would be expected if chronic dry-eye type symptoms after LASIK are manifestations of persistent neuropathic ocular pain.

Risk factors for the development of PPP after operations such as amputation, breast surgery, thoracotomy, herniorrhaphy, coronary artery bypass, and cesarean section include female gender (where applicable), younger age, presence and severity of persistent pre-operative pain, severity of post-operative pain, type of surgery, and genetic factors [52]. Many similar risk factors have also been found in dry eye after LASIK and dry eye in general. A study examining risk factors for the development of dry eye after LASIK found female sex, higher refractive correction, and greater ablation depth to be risk factors for the development of dry eye at 6 and 12 months post-operation [14], however another study did not identify gender as a risk factor [19]. Pre-existing dry eye symptomatology is another risk factor for severe or prolonged dry-eye following LASIK [6]. There is also evidence establishing the heritability of “primary” dry eye, that is, dry eye unrelated to ocular surgery. The genetics of susceptibility to post-operative dry eye/persistent ocular pain have yet to be extensively studied. However, functional variants in proinflammatory cytokines may be important in susceptibility to primary dry eye [14]. In addition to overlapping risk factors, the estimated incidence of chronic symptoms after LASIK fits nicely within the range expected of other PPP syndromes. Somewhere between 20-55% of LASIK patients will go on to develop at least mild symptoms of dry eye/persistent ocular pain, which overlaps with the expected incidence of PPP after breast surgery, coronary artery bypass surgery, amputation (30-50%), and thoracotomy (30-40%) [52-62].

As alluded to above, the epidemiology of PPP has been evaluated in a variety of surgical procedures and wide ranges have been found for its incidence depending on the surgery and method of ascertainment (Table 3) [55,59,61,63-70]. Certain surgeries, such as mastectomy and thoracotomy, have also been found to have a higher incidence of PPP. Thoracotomy in particular has been associated with extensive intercostal nerve damage, the degree of which has been found to correlate with the severity of PPP [52]. Since the development of PPP is generally related to nerve injury, it seems reasonable that surgical techniques to minimize nerve damage may reduce its incidence. There are multiple examples of this [52]. Laparoscopic herniorrhaphy can reduce the risk of nerve damage and pain as compared to an open procedure [52,71]. Muscle sparing thoracotomy results in less nerve damage and a reduction in PPP incidence as compared to a posterolateral approach [52,72]. During mastectomy, it has also been observed that preservation of the intercostobrachial nerve reduces chronic pain incidence [52,67,73]. Likewise, there is evidence that the incidence of persistent post-LASIK ocular pain symptoms differs based on surgical technique. Location of the corneal flap hinge is one variable that has been investigated. Some studies report an increase in symptoms, along with reduced corneal sensation, with a superior as compared to nasal hinge location during the creation of the corneal flap [20,74]; however, others have found no differences by hinge position [31,33,75,76]. The rationale behind these investigations relates to corneal neuroanatomy: because the corneal nerves predominately enter the cornea at 3 and 9 o’clock positions, the nasal hinge transects just one of these areas, while the superior hinge severs both [20]. A 2012 meta-analysis of 8 randomized controlled trials (ranging from *n* = 35 to 212) found that hinge location may have some effect on early post-operative dry eye and corneal sensation, but found no difference by 6 months post-operatively [77]. The use of femtosecond laser versus keratome for corneal flap creation has also been investigated. One study of 183 patients randomized to either microkeratome of femtosecond laser found the incidence of dry eye signs and symptoms to be significantly lower in the laser group, however more recent, smaller studies have found no difference [78-80]. Surgical optimization to prevent corneal nerve injury (to the extent possible) due to LASIK remains an open question.

**Table 3:** **Incidence of persistent post-surgical pain after different surgical interventions**

**Study**	**Surgery**	***N***	**Design**	**Definition**	**Incidence**
Brandsborg 2007 [65]	C-section	1173	Questionnaire 1 year after surgery	Pain	31.9% at 1 year
Brander 2003 [64]	Total knee replacement	116	Prospective longitudinal	Significant pain (VAS > 40)	13.1% at 1 year
Inaba 2012 [59]	Inguinal hernia repair	191	Questionnaire	Any pain	14.7% at variable time points least 3 months after surgery
Oberg 2005 [61]	Laparoscopic inguinal herniorrhaphy	161	Questionnaire	Chronic pain; unclear definition	4% at variable time points
Duale 2014 [67]	Elective C-section, inguinal herniorrhaphy, breast cancer surgery, cholecystectomy, saphenectomy, sternotomy, thoracotomy, or knee arthroscopy	2397	Multicenter prospective longitudinal	4 items positive on DN4	All (20.6%); laparoscopic herniorrhaphy (3.2%); knee arthroscopy (15.8%); C-section (24.5%); thoracotomy (32.7%); breast cancer surgery (37.1%) at 6 months
Ilfeld 2014 [68]	Mastectomy	30	Prospective longitudinal	Brief pain inventory pain induced dysfunction	47% at 12 months
Liang 2013 [69]	Ventral hernia repair	122	Retrospective	Unclear definition categorical variable	17.2% at variable time points
Nikolajsen 2004 [70]	C-section	220	Questionnaire mean follow-up time 10.2 months after surgery	Pain at the time of the questionnaire	12.3% at variable time points

Difference in morbidity associated with LASIK versus photorefractive keratectomy (PRK) has also been investigated. PRK is a similar procedure to correct for refractive error; however, unlike LASIK, it does not utilize a corneal flap and the corneal surface is ablated directly to reach the stroma beneath. A prospective study published in 2000 demonstrated a greater reduction in tear secretion in LASIK versus PRK patients at 6 months post-operatively, however this study looked only at objective signs and not symptoms of ocular pain [81]. A more recent study prospectively compared the incidence of dry eye symptoms in 34 patients with one eye receiving LASIK and the other PRK, concluding that at 12 months there was no increase in dry eye symptoms over pre-operative baseline in either group. However, although uniquely controlled with the use of both techniques in each patient, with a sample size of only 34, it is not clear that the study is sufficiently powered to draw conclusions regarding the incidence of dry eye [82].

### Neuropathic ocular pain after LASIK: implications for prevention and treatment of persistent symptoms

If one accepts the idea that neuropathic ocular pain at least partially underlies persistent dry eye symptoms after LASIK, this opens up a new realm of possibilities for prevention and treatment. Since, as far as we are aware, no studies have been conducted to comprehensively evaluate pharmacological treatment efficacy for the prevention of persistent ocular pain following LASIK in humans, we rely on what is known about the treatment of PPP in other areas of the body, and will mention relevant studies applying these well-established findings to the prevention of neuropathic pain in the eye.

Gabapentin and pregabalin (referred to collectively as “gabapentinoids”) are mainstays in the treatment of neuropathic pain. As discussed previously, the modulation of sensitization processes in the dorsal horn is a key target for pharmacologic treatment of PPP. The gabapentinoids bind to the regulatory alpha-2 delta (α2δ) subunit of N-type voltage gated calcium channels in dorsal root ganglia, in the dorsal horn and periaqueductal gray, with the ultimate effect of reducing excitatory neurotransmission [47,83]. There is also evidence for antagonism of the NMDA receptor, which, as previously discussed, may play a role in the process of central sensitization. In addition to these central actions, gabapentin has also been shown to reduce discharge from injured peripheral nerves [84].

Schmidt et al. nicely summarized that gabapentin has been shown to decrease the incidence of PPP after many but not all surgical procedures in randomized, placebo-controlled trials [85]. Surgeries where gabapentinoids had a positive effect included abdominal hysterectomy, herniorrhaphy, thyroidectomy, mastectomy, knee arthroplasty, cardiac surgery, and lumbar discectomy [85]. Dosing strategies ranged from high dose pre-operative administration only, to a low pre-operative dose followed by an extended taper [85-88] (Table 4).

**Table 4:** **Prevention of chronic post-operative pain with perioperative gabapentin and pregabalin: randomized, placebo-controlled studies**

**Study**	**Surgery**	**Metric of pain assessment**	**Treatment**	**Outcome**
Fassoulaki 2005 [86]	Breast surgery for cancer	Presence of pain (yes vs no)	Gabapentin (400 mg q6h, started 12 h pre-op, continued to POD 8, local anesthetic cream, and ropivacaine in wound (n = 25) vs placebo (n = 25)	Non-significant trend to less pain (30% vs 57%) and anesthetic use (0% vs 19%) at 6 months
Buvanendran 2010 [87]	Total knee arthroplasty	Neuropathic pain via Leeds Assessment of Neuropathic Symptoms and Signs scale	Pregabalin (300 mg) (n = 113) vs placebo (n = 115)	Significantly reduced pain in pregabalin group (0% at 3 & 6 months) vs placebo (8.7% and 5.2% at 3 & 6 mo).
Sen 2009 [88]	Elective hysterectomy	Verbal rating scale scores	Gabapentin 1.2 g w/placebo infusion (n = 20); ketamine w/oral placebo (n = 20); Control group (n = 20)	Significantly reduced pain scores in gabapentin group vs ketamine & placebo at 6 months

Regarding LASIK and the related vision correction procedure, PRK, the gabapentinoids have only been studied in the context of short term post-operative pain, and have been shown to decrease immediate post-operative pain following PRK in 2 of the 3 [89-92] prospective, randomized control trials to date. The largest of these studies (*n =* 150) demonstrated the positive effect of the gabapentinoids and demonstrated the equivalent effectiveness of gabapentin and pregabalin. However, these studies of immediate post-operative pain would not capture the later effects of this drug, and it is important to note that many studies of gabapentinoids in PPP prevention find this class more effective at reducing the risk of late but not immediate post-operative pain [86,93].

In addition to the gabapentinoids, opioids, alpha-2 adrenergic agonists, anti-depressants (specifically tricyclic and serotonin norepinephrine reuptake inhibitor classes of anti-depressants), and NMDA antagonists (ketamine) have also been used with success to treat chronic neuropathic pain. Local anesthetics have also been shown to reduce PPP by blocking peripheral input to the central nervous system [47].

Several topical agents, both targeting nerve growth and inhibiting inflammation, have been found to improve corneal sensitivity and nerve regeneration after LASIK in animal models. For example, topical insulin-like growth factor-1 (IGF-1) had positive effects on corneal surface ultrastructure, nerve regeneration, and tear parameters compared to controls in a rabbit model of LASIK [94]. In a similar model, both macrophage migration inhibitory factor and a combination of nerve growth factor (NGF), neurotrophine-3, interleukin-6, and leukemia inhibitory factor improved corneal sensitivity (via Cochet-Bonnet esthesiometer) in a more rapid manner compared to controls [95]. Another rabbit study found that eyes treated with NGF demonstrated an earlier and faster recovery of corneal sensitivity after LASIK compared to balanced salt treated controls [96]. These agents have not been evaluated in humans, however a 2005 study of 35 patients did find a correlation between higher levels of tear fluid NGF and improved corneal sensation in PRK as opposed to LASIK patients [97].

Autologous serum eye drops have been used for tear replacement in patients with severe dry eye. These tears are believed to be enriched with neurotrophic factors that could aid in nerve healing, including NGF [98]. Despite the theoretical potential of this treatment, in a prospective, randomized study of 54 eyes (27 male patients) undergoing LASIK, although improvement of some ocular signs of dry eye were reported in the experimental group, no differences were noted in the subjective scores for dryness between patients using autologous serum eye drops post-operatively compared to artificial tears groups [99]. However, it bears mentioning that at this sample size, this study is underpowered to detect anything other than a very large effect size, and a larger study would be needed to detect mild to moderate improvement.

Moving forward, LASIK offers an ideal elective surgical model in which to study the factors influencing the development of PPP. The benefits of this model would be multiple. Because of the elective nature of the procedure, we know when the surgery is going to occur, allowing us to monitor both pre- and post-operatively for environmental and genetic risk factors (such as dry eye symptoms, central sensitivity, or chronic pain syndromes), as well as perform corneal, facial, and generalized quantitative sensory testing before and after surgery, allowing us to define the natural history of disease development in susceptible individuals. The elective nature of the procedure (and the frequency with which it is performed) also facilitates the design of randomized controlled trials of reasonable size and appropriate power to detect differences due to risk factors, the efficacy and safety of potential treatments, and the pharmacogenomics of treatment response. Additionally, LASIK is generally performed according to a standardized protocol where nerve damage is expected to be reproducible. The corneal sub-basal nerve plexus is also uniquely accessible to the researcher, and may be easily visualized with confocal microscopy.

### Shared susceptibility factors may underlie persistent post-surgical pain, including LASIK

Virtually all disease arises from the interaction between genetic susceptibility and environmental factors, and the emergence of persistent ocular pain following refractive surgery is likely no different. Potential environmental triggers influencing outcomes may include surgical approach, including hinge location and size, type of procedure (LASIK versus PRK), and even, as some experts have suggested, stress and chemical exposure (such as that to alcohol, drugs, and environmental toxins) [100]. Genetics is also believed to play an important role in determining the clinical variability observed in nociception, pain processing, and therapeutic response [101,102]. These genetic factors may represent a blueprint for predisposition to chronic pain syndromes, including PPP as it manifests after LASIK.

Functional DNA variants (genetic polymorphisms) are known to impact neuronal function and inflammation, and are therefore likely to modulate the clinical presentation of PPP after LASIK and affect symptom onset and severity. While there are only a few reports on genetic polymorphisms and symptoms of primary DE, substantial research supports the fact that relatively common inherited genetic polymorphisms underlie individual differences in pain perception [102], pain related behaviors and the development of persistent pain syndromes [100,103]. For example, COMT (Catechol-O-methyl transferase) is an important gene whose functional variants have been described in over 100 publications and 30 reviews. COMT variants are associated with various forms of pain including neuropathic pain, post-surgical pain, and post-surgical pain severity [104-110]. Other gene variants found to be associated with PPP include the HLA genotype DRB1*04 and DQB1*03:02 allele, both of which are associated with chronic pain after inguinal hernia repair (n = 189) and lumbar disc herniation (n = 258) [111]. Genetic variants in voltage-gated sodium channels, GTP cyclohydrolase and tetrahydrobiopterin have also demonstrated an association with PPP [47].

In another study, 1 cytokine gene polymorphism (interleukin [IL] 1 receptor 2 single nucleotide polymorphism [SNP] rs11674595) and 1 haplotype (IL10 haplotype A8) were associated with PPP after breast cancer surgery [112]. Inflammation is also an important component of dry eye and polymorphisms in the proinflammatory cytokine genes IL-1β (SNP rs1143634) and IL-6R (SNP rs8192284) have been reported to associate with non-Sjogren dry eye symptoms in a Korean population [113]. This finding makes intuitive sense, as inflammation of the ocular surface together with a genetic propensity for inflammation can profoundly influence neuroplasticity and contribute to neuronal dysfunction and neuropathic pain [114-116]. With only limited information available, more research is needed on genetic susceptibility and epigenetic alterations associated with the severity and persistence of dry eye symptoms, including those emerging after LASIK.

Recently, a large twin study demonstrated that shared genetic factors underlie a number of comorbid chronic pain conditions, including chronic widespread musculoskeletal pain, pelvic pain, irritable bowel syndrome, and dry eye, and that this latent genetic factor had an estimated heritability of 66%. This finding supports the notion of common pathways of susceptibility and an underlying genetic disposition to developing chronic pain [117]. Our own data further expands on this by demonstrating that presence of neuropathic ocular pain (again, as manifests with “dry eye” type symptoms) in an individual is strongly associated with the presence of 3 or more comorbid pain disorders (unpublished observations of 115 patients with mild to moderate dry eye). With these studies, we have just begun to elucidate the mechanisms of heritable predisposition to the development of these syndromes including dry eye. Genomic studies to identify functional variants in genes and biologic pathways critical to disease susceptibility and resistance will be critical moving forward, with the hope that these types of studies will allow us to better understand the mechanism of the development of PPP syndromes using LASIK as a prototype, and potentially lead to the discovery of preventive approaches and mechanism-based treatments.

## Conclusions

With this review we have attempted to make three major points. First, that perhaps the experience of persistent dry eye symptoms following LASIK is better conceptualized as a neuropathic ocular surface pain syndrome involving mechanisms of both peripheral and central sensitization. Second, given this and the well-established evidence regarding injury to corneal nerves during the LASIK procedure, we may look to other PPP syndromes as the prototype for the management of persistent neuropathic ocular pain following LASIK procedures, and should revisit the use of existing neuromodulators for prevention and treatment in well-controlled randomized trials that are appropriately powered to assess efficacy and safety of known and novel approaches. Third, that the development of PPP in the eye or anywhere else in the body is the product of the interaction of genetic and environmental factors, and that understanding the genetics of susceptibility to these disorders will be important in realizing novel preventative and mechanism based treatments. We believe that it is important to better define the role of anti-neuropathic pain treatment in modulating ocular sensory apparatus function in patients with this symptom complex, and to integrate this tactic into a multi-modal approach including the treatment of ongoing ocular surface damage with ocular surface protection and anti-inflammatory agents. Furthermore, agents such as the gabapentinoids, may be promising in reducing the likelihood of development of central sensitization by attenuating the afferent trafficking of pain signals and excitatory synaptic transmission.

## Abbreviations

LASIK: Laser in-situ keratomileusis
PPP: Persistent post-operative pain
NMDA: N-methyl-D-aspartate
PRK: Photorefractive keratectomy
NGF: Nerve growth factor
COMT: Catechol-O-methyl transferase
SNP: Single nucleotide polymorphism
